# Supplementary figures and images for: Pairing Mouse Social and Aversive Stimuli across Sexes Does Not Produce Social Aversion in Females
Source: eNeuro. 2025 Dec 17;12(12):ENEURO.0228-25.2025. doi: 10.1523/ENEURO.0228-25.2025 (PMC12721876; doi:10.1523/ENEURO.0228-25.2025)

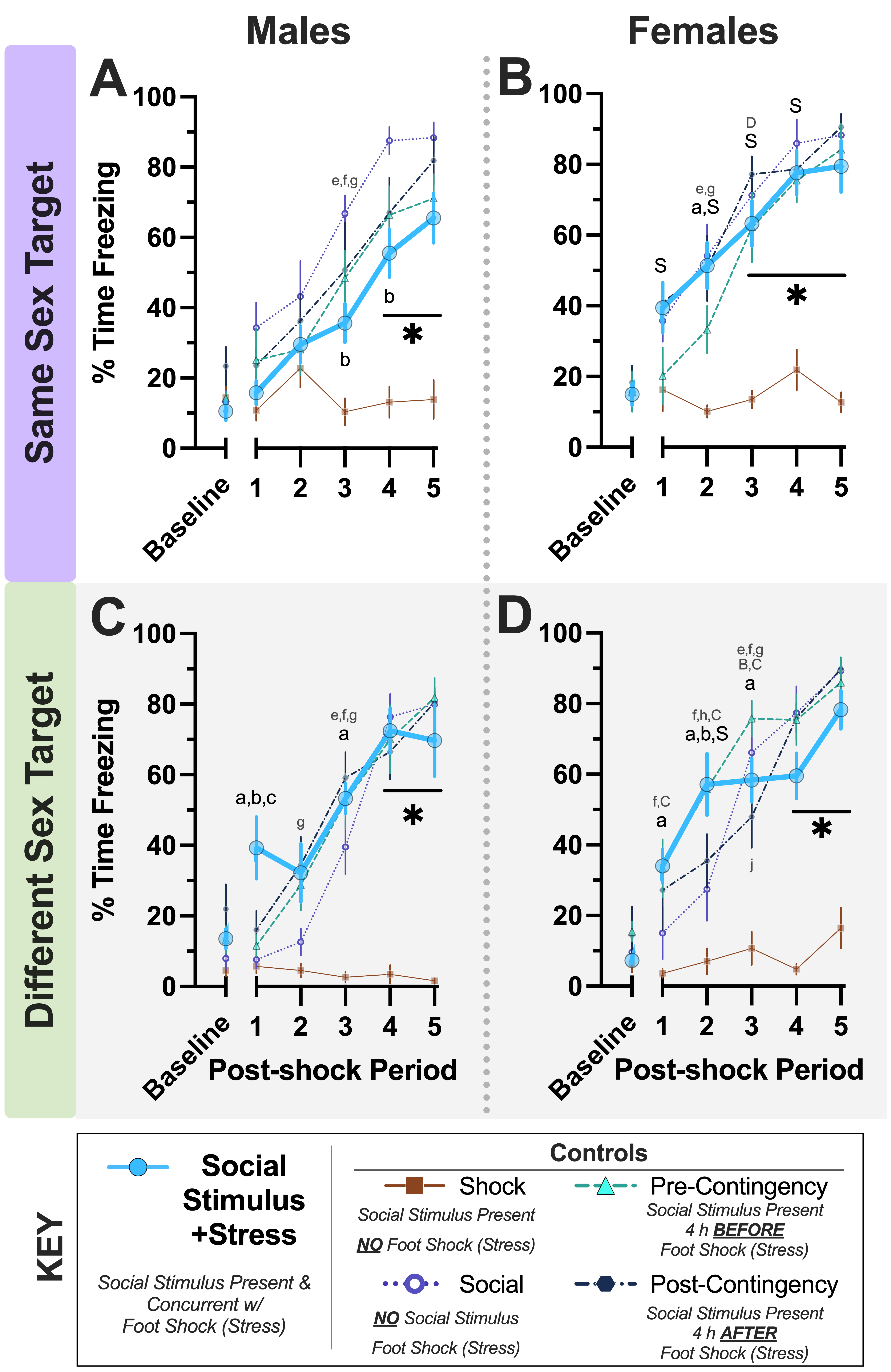

Supplement: Figure 2-1 — Day 1 acquisition during social conditioning procedure. Percent time freezing during social conditioning acquisition for mice in same sex (Panels A, B) and different sex (Panels C, D) experiments for male (A, C) and female (B, D) mice. Numbers of mice graphed within Panels A-D in order: Social Stimulus + Stress (n = 8, 9, 9, 8); Shock Control (8, 8, 7, 7); Social Control (n = 8 for all); Pre-Contingency Control (n = 7, 8, 8, 9); Post-Contingency Control (n = 7, 8, 8, 8). Average freezing for the first two minutes, prior to commencement of acquisition, is plotted on the x-axis as baseline. The average percent freezing for each 30 second period following each of the five mild foot shocks are thereafter plotted along x-axis (Post-shock Periods 1-5). Alphabetically, within experiment, sex, and time point: aindicates (Shock Control vs. Social Stimulus + Stress) p < 0.001, p = 0.007, p < 0.001, p = 0.025, p < 0.001, p < 0.001. bindicates (Social Control vs. Social Stimulus + Stress) p = 0.026, p = 0.005, p = 0.009, p = 0.038. cindicates (Pre-Contingency Control vs. Social Stimulus + Stress) p = 0.032. eindicates (Shock Control vs. Social Control) p < 0.001, p < 0.001, p = 0.003, p < 0.001. findicates (Shock Control vs. Pre-Contingency Control) p = 0.004, p < 0.001, p = 0.024, p < 0.001, p < 0.001. gindicates (Shock Control vs. Post-Contingency Control) p = 0.002, p = 0.002, p = 0.047, p < 0.001, p = 0.002. hindicates (Social Control vs. Pre-Contingency Control) p = 0.043. jindicates (Pre-Contingency Control vs. Post-Contingency Control) p = 0.028. ✱indicates p < 0.001 Social, Pre-Contingency, and Post-Contingency Controls and Social Stimulus + Stress mice vs. Shock Controls. Alphabetically, within experiment, group, and time point: Bindicates (Social Controls male vs. female) p = 0.005. Cindicates (Pre-Contingency Controls male vs. female) p = 0.019, p = 0.006, p = 0.012. Dindicates (Post-Contingency Controls male vs. female) p = 0.012. Sindicates (Social Stimulus + Stres [file eneuro-12-ENEURO.0228-25.2025-s002.tif]

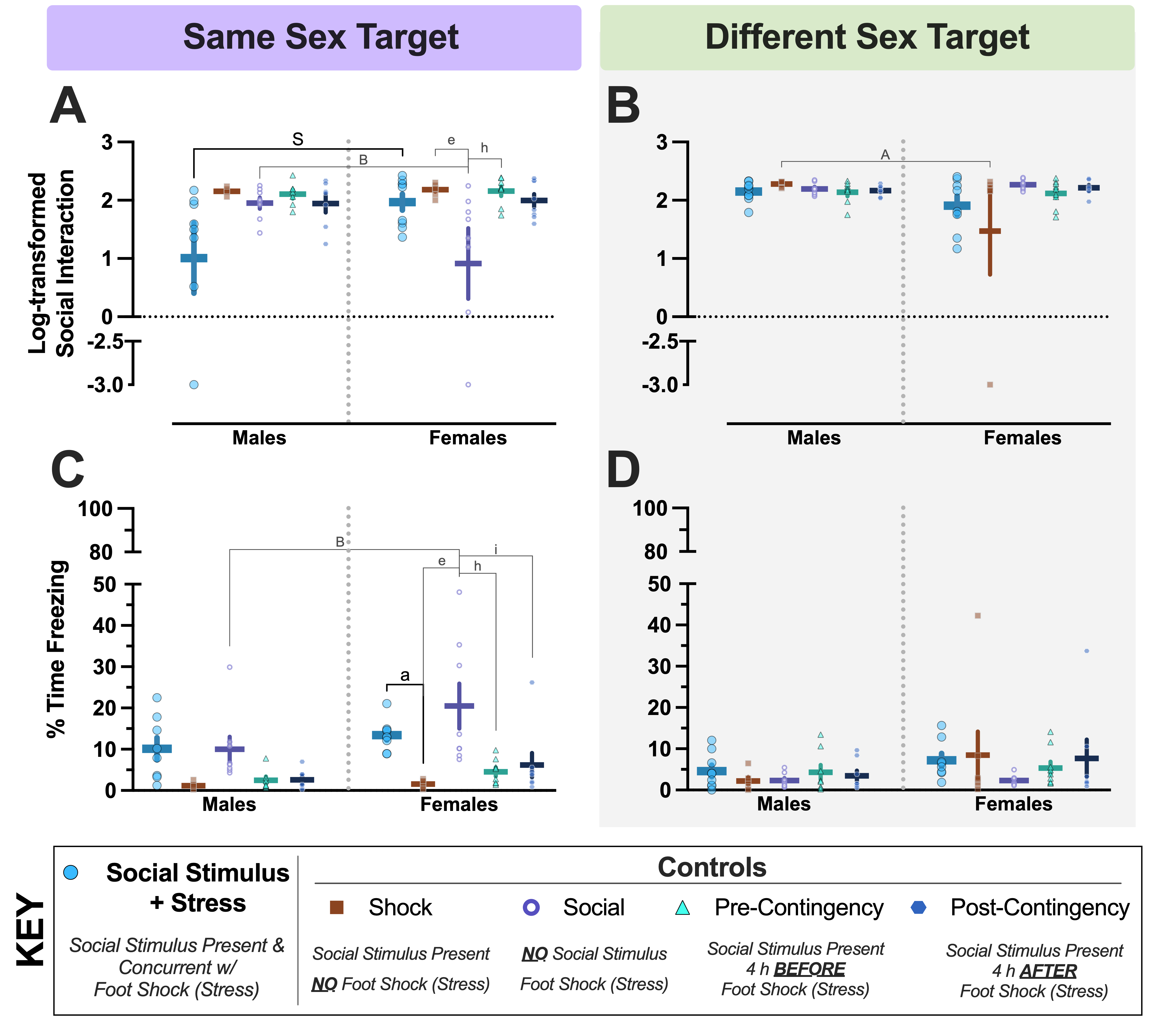

Supplement: Figure 3-1 — Day 2 social interaction and fear behaviors following social conditioning. Log-transformed social interaction (Panels A, B) and percent time freezing during post-test social interaction (Panels C, D) data are shown for mice in the same (A, C) and different (B, D) sex experiments. Numbers of mice graphed within each panel, left to right: A) n = 8, 7, 8, 7, 7, 9, 8, 8, 8, 8; B) n = 9, 6, 8, 8, 8, 8, 7, 7, 9, 7; C) n = 8, 7, 8, 7, 7, 9, 8, 8, 8, 8; D) n = 9, 6, 8, 8, 8, 8, 7, 7, 9, 7. Y axes for C, D were split to facilitate clearer visualization of the low freezing levels exhibited during social engagement testing. Left to right, top to bottom: Sp = 0.018, Bp = 0.013, ep = 0.027, hp = 0.032, Ap = 0.023 [authors’ note: we consider this a false positive], Bp = 0.004, ap = 0.008, ep < 0.001, hp < 0.001, iindicates (Social Control vs. Pre-Contingency Control) p = 0.001. Data graphed as mean ± standard error of the mean. Download Figure 3-1, TIF file. [file eneuro-12-ENEURO.0228-25.2025-s004.tif]

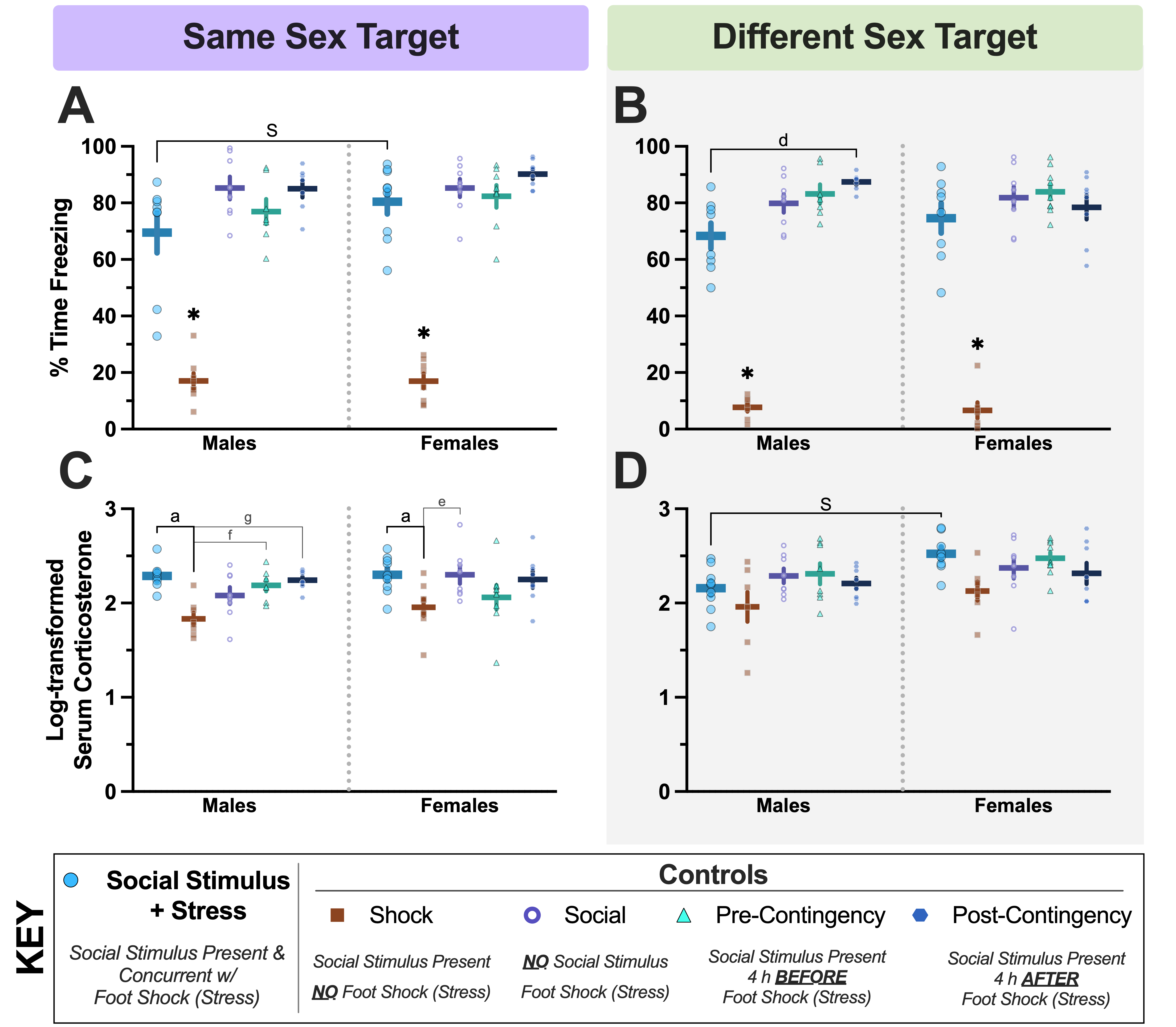

Supplement: Figure 4-1 — Day 3 social conditioning context fear expression averages and subsequent log-transformed serum corticosterone levels. Average context fear behavior (Panels A, B) and log-transformed serum corticosterone levels (Panels C, D) data are shown for mice in all groups in the same sex (A, C) and different sex (B, D) experiments. Numbers of mice graphed within Panels A-D in order, left to right: A) n = 8, 8, 8, 7, 7, 9, 8, 8, 8, 8; B) n = 8, 7, 8, 7, 7, 8, 7, 8, 9, 8; C) n = 7, 8, 8, 7, 7, 9, 8, 8, 8, 8; D) n = 9, 7, 8, 8, 8, 8, 7, 8, 9, 8. Omnibus statistics for data in Panel C: Group × Sex - F(4,68) = 1.207, p = 0.316, partial η2 = 0.066; Group - F(4,68) = 7.098, p < 0.001, partial η2 = 0.295; Sex - F(1,68) = 0.813, p = 0.370, partial η2 = 0.012; one male same sex Social Stimulus + Stress mouse was excluded as an outlier (>4 standard deviations less than the mean). Omnibus statistics for data in Panel D: Group × Sex - F(4,70) = 0.709, p = 0.589, partial η2 = 0.039; Group - F(4,70) = 3.841, p = 0.007, partial η2 = 0.180; Sex - F(1,70) = 8.708, p = 0.004, partial η2 = 0.111. Left to right, top to bottom: Sp = 0.049, dindicates (Pre-Contingency Control vs. Social Stimulus + Stress) p = 0.004, ap = 0.004, fp = 0.045, gp = 0.012, ap = 0.033, ep = 0.044, Sp = 0.007. ✱p < 0.001 Social, Pre-Contingency, and Post-Contingency Controls and Social Stimulus + Stress mice vs. Shock Control. Data graphed as mean ± standard error of the mean. Download Figure 4-1, TIF file. [file eneuro-12-ENEURO.0228-25.2025-s003.tif]
