# Supplementary material for: Pairing Mouse Social and Aversive Stimuli across Sexes Does Not Produce Social Aversion in Females
Source: eNeuro. 2025 Dec 17;12(12):ENEURO.0228-25.2025. doi: 10.1523/ENEURO.0228-25.2025 (PMC12721876; doi:10.1523/ENEURO.0228-25.2025)
Supplement: Table 5-2 — Outlier identification for female Post-Contingency Control mice in the Different Sex experiment after log-transforming social interaction. Download Table 5-2, DOCX file. [file eneuro-12-ENEURO.0228-25.2025-s008.docx]

Extended data Table 5-2 for

**Pairing mouse social and aversive stimuli across sexes does not produce social aversion in females**

Abbreviated title: **Sex effects on social conditioning and behavior**

Jasmin N. Beaver^a,b,c^, Marissa M. Nicodemus^a,b^, Isabella R. Spalding^a^, Lauren R. Scrimshaw^a,b^, Sohini Dutta^b,d^, Aaron M. Jasnow^e^, Lee M. Gilman^a,b,c^*

^a^Department of Psychological Sciences, Kent State University, Kent, OH, USA 44242

^b^Brain Health Research Institute, Kent State University, Kent, OH, USA 44242

^c^Healthy Communities Research Institute, Kent State University, Kent, OH, USA 44242

^d^School of Biomedical Sciences, Kent State University, Kent, OH, USA 44242

^e^Department of Pharmacology, Physiology, and Neuroscience, University of South Carolina School of Medicine, Columbia, SC, USA 29209

Author contributions: JNB, SD, AMJ, LMG designed research. JNB, MMN, IRS, LRS, and LMG performed research. LMG contributed analytic tools. JNB and LMG analyzed data and wrote the manuscript. All authors reviewed and approved the manuscript.

Correspondence should be addressed to:

Lee M. Gilman, Ph.D.

lgilman1@kent.edu

600 Hilltop Dr.

209 Kent Hall

Kent State University

Kent, OH, USA 44242

Acknowledgements: We gratefully acknowledge the mice used in this study, the unrivaled veterinary care by Stan Dannemiller, DVM, MS, DACLAM, and the dedicated work of our vivarium caretakers. Figure 1 was created with BioRender.com (Toronto, ON).

Conflict of Interest: The authors declare no competing financial interests.

Funding sources: This work was supported by R15 MH118705 to AMJ and LMG, and by Kent State University.

If manuscript is accepted for publication, raw data will be made available at:

<https://osf.io/mazfx/?view_only=e6a17be6021d4c80a440e2f9b0ce8d74>

Table 5-2. Outlier identification for female Post-Contingency Control mice in the Different Sex experiment after log-transforming social interaction.

| **Log-Transformed Social Interaction** | | | |
| --- | --- | --- | --- |
| Different Sex | Females | Social Controls | |
| **Mouse ID** | **Data** | **Outlier Determination** | |
| SB104 | 2.372 | *2.214* | *Avg* |
| SB114 | 2.187 | *0.134* | *SD* |
| SB119 | 2.240 | *1.678* | *Avg - 4 SD* |
| SB120 | 1.569 |  |  |
| SB159 | 1.976 |  |  |
| SB160 | 2.154 |  |  |
| SB161 | 2.360 |  |  |
| SB162 | 2.210 |  |  |
